# Intraoperative fluid optimization using stroke volume variation in high risk surgical patients: results of prospective randomized study

**DOI:** 10.1186/cc9070

**Published:** 2010-06-16

**Authors:** Jan Benes, Ivan Chytra, Pavel Altmann, Marek Hluchy, Eduard Kasal, Roman Svitak, Richard Pradl, Martin Stepan

**Affiliations:** 1 Department of Anesthesiology and Intensive Care, Charles University teaching hospital, alej Svobody 80, Plzen, 304 60, Czech Republic

## Abstract

**Introduction:**

Stroke volume variation (SVV) is a good and easily obtainable predictor of fluid responsiveness, which can be used to guide fluid therapy in mechanically ventilated patients. During major abdominal surgery, inappropriate fluid management may result in occult organ hypoperfusion or fluid overload in patients with compromised cardiovascular reserves and thus increase postoperative morbidity. The aim of our study was to evaluate the influence of SVV guided fluid optimization on organ functions and postoperative morbidity in high risk patients undergoing major abdominal surgery.

**Methods:**

Patients undergoing elective intraabdominal surgery were randomly assigned to a Control group (n = 60) with routine intraoperative care and a Vigileo group (n = 60), where fluid management was guided by SVV (Vigileo/FloTrac system). The aim was to maintain the SVV below 10% using colloid boluses of 3 ml/kg. The laboratory parameters of organ hypoperfusion in perioperative period, the number of infectious and organ complications on day 30 after the operation, and the hospital and ICU length of stay and mortality were evaluated. The local ethics committee approved the study.

**Results:**

The patients in the Vigileo group received more colloid (1425 ml [1000-1500] vs. 1000 ml [540-1250]; *P *= 0.0028) intraoperatively and a lower number of hypotensive events were observed (2[1-2] Vigileo vs. 3.5[2-6] in Control; *P *= 0.0001). Lactate levels at the end of surgery were lower in Vigileo (1.78 ± 0.83 mmol/l vs. 2.25 ± 1.12 mmol/l; *P *= 0.0252). Fewer Vigileo patients developed complications (18 (30%) vs. 35 (58.3%) patients; *P *= 0.0033) and the overall number of complications was also reduced (34 vs. 77 complications in Vigileo and Control respectively; *P *= 0.0066). A difference in hospital length of stay was found only in per protocol analysis of patients receiving optimization (9 [8-12] vs. 10 [8-19] days; *P *= 0.0421). No difference in mortality (1 (1.7%) vs. 2 (3.3%); *P *= 1.0) and ICU length of stay (3 [2-5] vs. 3 [0.5-5]; *P *= 0.789) was found.

**Conclusions:**

In this study, fluid optimization guided by SVV during major abdominal surgery is associated with better intraoperative hemodynamic stability, decrease in serum lactate at the end of surgery and lower incidence of postoperative organ complications.

**Trial registration:**

Current Controlled Trials ISRCTN95085011.

## Introduction

Fluid administration in the intraoperative period is an integral part of everyday anesthesiology practice. Adequate intravascular volume replacement is a crucial issue that can seriously affect postoperative organ function and hence outcome [1-3]. Guiding fluid management using standard physiologic variables (blood pressure, heart rate etc) may be associated with a state of occult hypoperfusion [4]. Goal-directed therapy (GDT) was proposed to overcome this problem by introducing different hemodynamic variables into a dynamic perspective of individualized fluid loading and use vasoactive substances to reach predefined goal of optimal preload and/or oxygen delivery [5,6].

In past years, many trials using different devices and goals have been published in the literature demonstrating better outcomes in organ functions [7,8], morbidity [9-14] or even mortality [15]. Esophageal Doppler was used by many for guiding fluid management with good results but its use is partially limited by the need for deep sedation [16] and experienced staff [17]. Also, the reliability in major vascular procedures requiring cross-clamping of descendent aorta could be questioned. Different algorithms for arterial pressure wave analysis have been introduced lately. As arterial cannulation is routinely used for continuous blood pressure monitoring in high-risk patients, their use is not associated with increased invasivity and risk. These monitors are generally well tolerated by patients and easy to maintain. Some of these devices have already been used in GDT trials [12].

With the introduction of arterial pressure waveform analysis, the well-known interaction between stroke volume variation (SVV) and lung inflation during mechanical ventilation [18] has become available for routine clinical use. Several studies documented the usefulness of blood pressure variations and it surrogates (pulse pressure variation or systolic pressure variation) in predicting position on the Frank-Starling curve and hence fluid responsiveness [19-22]. The reliability of automated assessment [23], the influence of ventilator setting [24,25] and afterload modification [26] were also addressed in the literature.

Vigileo/FloTrac is a continuous monitor of patient's hemodynamic status on a beat-to-beat basis using online analysis of arterial pressure waveform. Cardiac performance is calculated assessing the arterial tree impedance (defined as coefficient Khi -χ), so no external calibration is needed and the device is ready to use after obtaining basic demographic parameters [27]. In past years, the arterial impedance calculation was criticized and use in clinical practice was debatable [28-30]. However, with software modifications and more frequent calculation of impedance, the device accuracy improved [31,32,35]. Despite some controversy [36,37] it is used in clinical practice. SVV derived by Vigileo/FloTrac has shown good correlation with results acquired from the PiCCO system and with a cut-off value of 9.6% a good sensitivity and specificity for predicting fluid responsiveness [21]. The aim of our prospective randomized study was to examine the effect of SVV-guided fluid therapy in the perioperative care of high-risk surgical patients and its influence on postoperative morbidity and mortality in comparison with standard treatment.

## Materials and methods

This was a prospective, randomized, partially blinded, single-center study conducted between July 2007 and May 2009 at the Department of Anesthesiology and Intensive Care Medicine, at Charles University Teaching Hospital in Plzeň. The trial was approved by local research ethics committee and all patients gave their informed consent. High-risk patients scheduled for major abdominal surgery with anticipated operation time longer than 120 minutes or presumed blood loss exceeding 1,000 ml (i.e. colorectal or pancreatic resections, intraabdominal vascular surgery) were screened for eligibility. At least one operation-related and one patient-related inclusion criteria had to be fulfilled (Table 1). Patients younger than 18 years, with documented arrhythmias and with a weight below 55 kg or above 140 kg were excluded to ensure accuracy of Vigileo/FloTrac measurement [38].

**Table 1:** Inclusion criteria

Procedure-related (at least one of them)
Operation duration more than 120 minutes and opened peritoneal cavity
Presumed blood loss more than 1,000 ml

**Patient related (at least one of them)**
Ischemic heart disease or severe heart dysfunction
Chronic obstructive pulmonary disease (moderate to severe)
Age above 70 years
ASA 3 or more for other reasons (chronic kidney disease, diabetes etc.)

**Exclusion criteria**
Irregular heart rhythm
Body weight less than 55 kg or more than 140 kg
Age under 18 years

ASA, American Society of Anesthesiologists' physical status classification.

### Patients randomization and outcome measures

**Figure 1:**
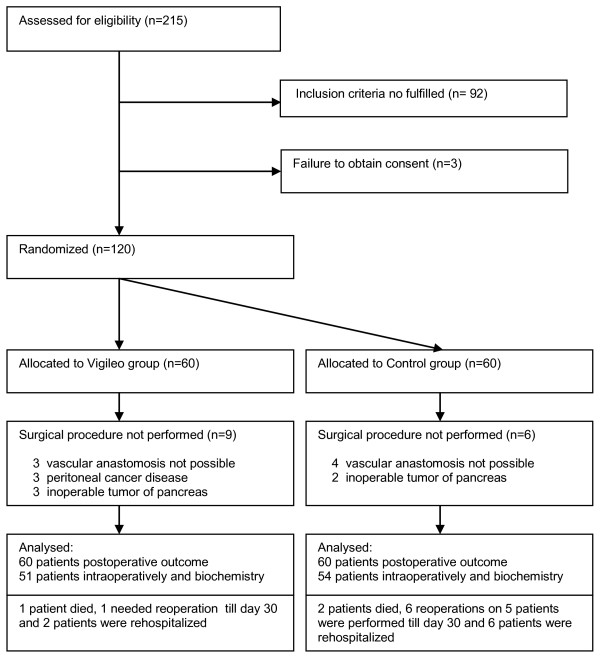
**Flow of participants through the trial**.

Patients meeting inclusion criteria were randomized using opaque sealed envelopes to intervention (Vigileo) or control (Control) group. The anesthetist responsible for intra-operative management was aware of the group assignment, whereas all other members of research team, other health care providers and patients were not. Randomization concealment for researchers was broken only at the end of the study for statistical data analysis. In case of intraoperative change in procedure performed (abandoning planned surgery because of inoperability or performing just a minor procedure), study protocol optimization was not realized, but their postoperative outcome was assessed in the intention-to-treat analysis (Figure 1)

Primary outcome measures were postoperative morbidity based on number of infectious and other organ complications until day 30 after the operation, consistent with previous studies on peri-operative optimization [11,12,16]. Secondary outcome measures were hospital/ICU length of stay and all-cause mortality. These parameters were assessed both on intention-to-treat and per protocol basis. Biochemical parameters of oxygen debt during operation and in early postoperative period (8 hours), that is serum lactate level, central venous oxygen saturation (ScvO2), arterial acid-base balance parameters and intraoperative hemodynamic parameters and amounts of intravenous fluids and inotropes used were assessed only in per protocol patients.

### Peri-operative care

A central venous catheter was inserted via the subclavian or internal jugular vein the day before surgery. An anteroposterior chest x-ray was obtained to exclude catheter malposition. Patients were premedicated according to institutional standards and an infusion of balanced crystalloid solution (Ringerfundin; B.Braun Melsungen Ag, Melsungen, Germany) at a rate of 2 ml/kg/hr was started at 8 am on the day of surgery. Baseline demographic parameters, blood pressure, and heart and respiratory rates as well as preoperative Acute Physiologic and Chronic Health Evaluation II (APACHE II) and Sequential Organ Failure Assessment (SOFA) scores were recorded in the operating room. Before induction of anesthesia, an arterial line (20G, BD Arterial Cannula, BD Critical Care Systems Ltd., Singapore) was inserted into the radial artery of the non-dominant forearm and first measurements and laboratory blood were taken. Optimal pressure signal damping was assessed using flush test before the first measurements. In the patients, who gave informed consent for epidural analgesia, a catheter for postoperative pain management was inserted between the Th7/8 and L1/2 vertebral interspaces and after performing a test for correct extradural placement, a dose of sufentanil 10 ug in 10 ml saline solution was administered. Anesthesia was than induced using either thiopental 4 mg/kg, propofol 2 mg/kg or etomidate 2 mg/kg in combination with sufentanil 5 to 15 ug. Tracheal intubation was facilitated by neuromuscular relaxation (atracurium, cis-atracurium or rocuronium), depending on co-morbidities and anesthesiologists choice. Anesthesia was maintained with volatile anesthetics (sevoflurane or desflurane) in N _2_O and O _2 _mixture (0.9 to 1.2 MAC). Sufficient analgesia was provided using 5 ug boluses of sufentanil, or with a continuous infusion of sufentanil 10 ug and bupivacain 25 mg in 20 ml saline at a rate of 4 to 6 ml/hr via an epidural catheter. All patients were mechanically ventilated with tidal volume 8 ml/kg and positive end-expiratory pressure (PEEP) 0.6 kPa, respiratory rate was set to maintain normocapnia. Anemia (hemoglobin level below 90 g/l) and acute blood loss higher than 20% of calculated patient's circulatory volume were corrected with transfusions of packed red blood cells (RBC) and fresh frozen plasma (FFP), respectively. The number of transfused units (both RBC and FFP) was recorded as well as the amount of infused crystalloid and colloid solutions, diuresis and blood loss. At time of skin closure, blood was taken for acid-base balance analysis (both arterial and central venous), blood count and basic biochemical laboratory tests.

### Study protocol

**Figure 2:**
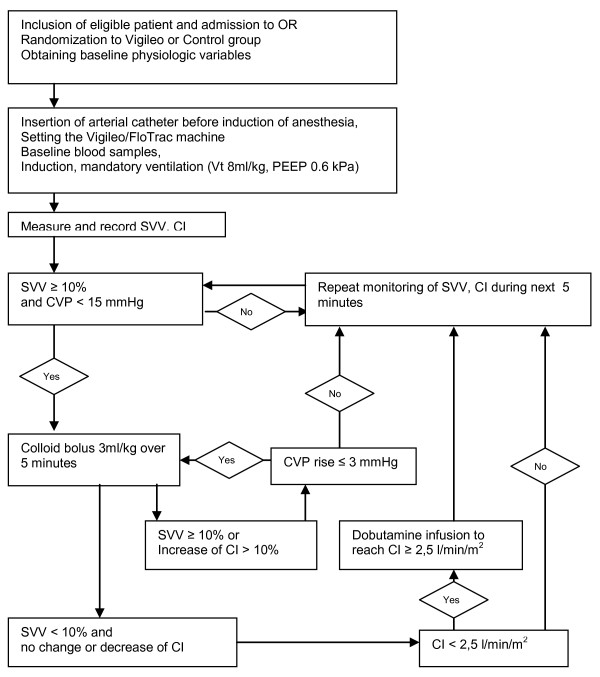
**Fluid management in Vigileo group**. CI, cardiac index; CVP, central venous pressure; SVV, stroke volume variation; PEEP, positive end-expiratory pressure; Vt, tidal volume; OR, operation room.

Vigileo/FloTrac device (Edwards Lifesciences, Irvine, CA, USA) with software version 1.10 was used for measuring SVV and other hemodynamic variables. Intraoperative basal fluid replacement was realized in both groups with continuous infusion of 8 ml/kg/hr of crystalloid solution (Plasmalyte; Baxter Czech spol.s.r.o, Praha, Czech Republic). In the Vigileo group, additional boluses of 3 ml/kg colloid solution (Voluven 130/0.4 6%; Fresenius Kabi AG, Bad Homburg, Germany, Tetraspan 130/0.4 6%; B.Braun Melsungen Ag, Melsungen, Germany) were given when SVV measured by Vigileo/FloTrac system rose above 10% (a sustained change during the previous five minutes) or in the case of positive response (cardiac index (CI) increase above 10%) to previous fluid challenge. Central venous pressure (CVP) measurement served as a regulatory mechanism (Figure 2). An infusion of dobutamine was started to maintain CI between 2.5 and 4 l/min/m ^2 ^under low cardiac output state conditions (CI less than 2.5 l/min/m ^2^) after appropriate fluid administration. Ephedrine boluses of 5 to 15 mg or norepinephrine infusion were allowed in addition to colloid infusion to treat a fall in systolic arterial pressure below 90 mmHg or mean arterial pressure (MAP) below 65 mmHg (e.g. during clamp release or sudden large blood loss etc.) to maintain MAP above 70 mmHg. These episodes were recorded as hypotensive events and underwent analysis. In the Control group, the anesthesiologist was free to give additional fluids (both crystalloids and colloids) or use vasoactive substances to maintain blood pressure, diuresis and CVP in normal ranges (MAP > 65 mmHg, heart rate < 100 bpm, CVP 8 to 15 mmHg, urine output > 0.5 ml/kg/h).

### Postoperative care and data collection

After surgery, the patients were transferred to either ICU or a monitored bed on the standard ward. During the postoperative period the patients were managed by an intensivist or a surgeon, who was not aware of the patient's allocation in study group. Biochemical tests (arterial and central venous blood gas, serum lactate, blood count and other laboratory tests) were performed at 4, 8 and 24 hours after the end of surgery. Basal measurements of blood pressure, heart and respiratory rates, peripheral hemoglobin oxygen saturation, diuresis, medication and intravenous fluids or blood products administered during eight hours postoperatively were retrospectively collected by a member of the research team blinded to patient allocation. Physiological and Operative Severity Score for the Enumeration of Mortality and Morbidity (POSSUM) [39] was calculated after operation along with SOFA score for the period of 8 and 24 hours postoperatively.

Patients were monitored up to discharge from the hospital for infectious and organ complications (cardiac, pulmonary, gastrointestinal, renal and thrombotic). The list of screened complications was based on the POSSUM scoring system and adapted according to other literature data [11,12,16]. Additionally, we followed complications deemed as life-threatening or disabling. Diagnosis and management of complications were undertaken by non-research staff. These were verified in accordance with predefined criteria [40,41] where available by a member of the research team unaware of patient group allocation. Complications that occurred after discharge and required ambulatory or in-hospital care up to day 30 after surgery were also recorded. The total number of complications and the number of patients with complications were calculated for each group. The ICU and standard care length of stay and length of ventilator support were recorded at the end of hospitalization. If a patient died, the time from operation to death was recorded.

### Statistical analysis

A high number of infectious and organ complications can be anticipated in high-risk surgical patients. According to our retrospective analysis of the incidence of complications in similar patient populations in our hospital (65%, unpublished data) and data from similar studies [8,11,12], the percentage of patients with postoperative complications can exceed 60% with a 50 to 60% reduction described in intervention groups. For a decrease in morbidity from 65 to 38%, a study sample size of 50 patients in each group was calculated for two-sided tests with type I error of 5% and power of 80%. Owing to an anticipated loss of 15 to 20% of patients entering the study due to a change in scheduled surgery, we proposed to include 60 patients in each group. For a test of normal distribution, the Kolmogorov-Smirnov test was used. Continuous data with normal distribution were tested with paired or unpaired t-tests, non-normally distributed data using Mann-Whitney U test and Wilcoxon rank-sum test for unpaired and paired results, respectively. The change in time-dependent variables was tested using analysis of variance (ANOVA) on repeated measurements or Friedman test. Categorical data were tested using chi-square test and chi-square test for trend. Unless stated otherwise, normally distributed data are presented as mean ± (standard deviation), and as median (interquartile ranges) where not normally distributed. A *P *< 0.05 was considered statistically significant for all tests. All calculations were performed with MedCalc^® ^version 10.4.8.0 (Frank Schoonjans, MedCalc Software, Broekstraat 52, 9030 Mariakerke, Belgium).

## Results

A total of 215 patients were found to be eligible according to scheduled surgical procedures from July 2007 to May 2009. After examining these patients for inclusion criteria and obtaining informed consent, 120 patients were included and randomized to the Vigileo or Control groups. Fifteen patients dropped out after randomization because of unanticipated cancellation of their surgery (nine patients from the Vigileo group and six patients from the Control group). There were no other discontinuations or patients lost to follow-up (Figure 1).

Both groups were equal in basic demographic parameters, co-morbidities, American Society of Anesthesiologists' physical status classification status or surgical procedure performed. No significant differences in basal scoring systems (APACHE II, SOFA and POSSUM scores) at baseline were observed. Patients were also comparable in terms of baseline biochemical laboratory parameters and physiologic variables (Table 2).

**Table 2:** Baseline demographics

Parameters	Vigileo group	Control group	*P *value
Number in group	60	60	
Male : Female	50 : 10	47 : 13	0.643
Age	66.73 ± 7.88	66.32 ± 8.38	0.78
Weight (kg)	80.47 ± 12.75	82.49 ± 17.18	0.466
Height (cm)	172.07 ± 7.2	172.1 ± 10.19	0.99
APACHE II score	6.59 ± 3.04	6.76 ± 2.61	0.758
SOFA score	1 (1-2)	1 (0-2)	0.82
POSSUM (operative score)	17 (16-20)	17 (14-20)	0.304
POSSUM (physiology score)	20 (19-23)	21 (19-23)	0.295
ASA (1:2:3:4:5)	0:14:37:9:0	0:11:40:9:0	0.646
Chronic disease			
Coronary artery disease	32 (53%)	31 (52%)	0.942
Hypertension	56 (93%)	56 (93%)	0.721
Peripheral artery disease	31 (52%)	30 (50%)	0.971
COPD/Asthma bronchiale	13 (22%)	12 (20%)	0.964
Other pulmonary pathology	5 (8%)	3 (5%)	0.767
Cerebrovascular disease	8 (13%)	8 (13%)	0.786
Diabetes mellitus	21 (35%)	23 (38%)	0.851
Chronic kidney disease	5 (8%)	4 (7%)	0.89
Malignancy	23 (38%)	23 (38%)	0.851
Age > 70 years	24 (40%)	21 (35%)	0.706
Surgical procedure			
Colo-rectal surgery	17 (28%)	16 (27%)	0.935
Pancreatic surgery	5 (8%)	3 (5%)	0.767
Intraabdominal vascular surgery	38 (63%)	41 (68%)	0.701
Surgery cancelled (Figure 1)	9 (15%)	6 (10%)	0.581
Epidural analgesia	35 (58%)	37 (62%)	0.794

Values are presented as absolute (percentage), mean ± standard deviation or median (interquartile range).

APACHE II, Acute Physiology And Chronic Health Evaluation score II; ASA, American Society of Anesthesiologists' physical status classification; COPD, chronic obstructive pulmonary disease; POSSUM, physiologic and operative severity score for the enumeration of mortality and morbidity score; SOFA, Sequential Organ Failure Assessment score.

The Vigileo group received a significant larger amount of colloid infusions (Vigileo 1,425 ml (1,000 to 1,500) vs. Control 1,000 ml (540 to 1,250); *P *= 0.0028), the volume of infused crystalloids, the amount of blood products and blood loss did not differ between the groups. There was a trend towards maintaining higher diuresis during the study period in the Vigileo group (1.13 (0.76 to 1.85) ml/kg/hr vs. 0.896 (0.56 to 1.43) ml/kg/hr in the Control group; *P *= 0.068). Lower incidence of intraoperative hypotensive events (2 (1 to 2) vs. 3.5 (2 to 6); *P *= 0.0001) and a trend toward lower use of norepinephrine (3 patients (5.88%) vs. 11 patients (20.37%); *P *= 0.058) was found in Vigileo group. The amount of fluids infused, diuresis, physiologic variables and pharmacological interventions within the first eight hours postoperatively did not significantly differ between the groups (Table 3). No difference in MAP, heart rate and CVP between the groups was observed at the end of surgery, although in both groups a significant decrease of MAP against baseline value was found. In the Vigileo group a decrease in heart rate (74 ± 13 vs. 70 ± 11; *P *= 0.0108), increase in CVP (8 ± 2 vs. 10 ± 3; *P *= 0.0002) from baseline occurred, while no such difference was observed in the Control group. At the end of surgery a decrease in SVV compared with preoperative value (13 ± 5 vs. 7 ± 2; *P *< 0.0001) was observed in the Vigileo group, a similar parameter was not evaluated in the Control group.

**Table 3:** Perioperative period ('per protocol' analysis)

Parameters	Vigileo group	Control group	*P *value
	n = 51 (85%)	n = 54 (90%)	
Baseline measurement			
MAP (mmHg)	103 ± 13	103 ± 16	0.948
CVP (mmHg)	8 ± 2	9 ± 4	0.362
HR (beats/min)	74 ± 13	74 ± 10	0.851
CI (ml/min/m^2^)	3 ± 0.64	N/A	
SVV (%)	13 ± 5	N/A	
			
End of surgery			
MAP (mmHg)	92 ± 12*	91 ± 14*	0.702
CVP (mmHg)	10 ± 3*	10 ± 3	0.439
	*(*P *= 0.0002 vs. baseline)		
HR (beats/min)	70 ± 11*	73 ± 15	0.264
	*(*P *= 0.0108 vs. baseline)		
CI (ml/min/m^2^)	3.6 ± 0.7*	N/A	
	*(*P* < 0.0001 vs. baseline)		
SVV (%)	7 ± 2*	N/A	
	*(*P* < 0.0001 vs. baseline)		
**Number of hypotensive periods intraoperatively**	**2 (1-2)**	**3.5 (2-6)**	**0.0001**
Length of anesthesia (min)	184 ± 46	202 ± 53	0.072
Length of surgery (min)	163 ± 44	176 ± 55	0.214
Length of aortic cross-clamping	61.5 ± 35	57 ± 35	0.592
Crystalloids (ml)	2321 ± 681	2459 ± 930	0.386
**Colloids (ml)**	**1425 (1000-1500)**	**1000 (540-1250)**	**0.0028**
Blood (ml)	0 (0-566)	270 (0-578)	0.633
Fresh frozen plasma (ml)	0 (0-540)	0 (0-540)	0.793
Estimated blood loss (ml)	700 (500-1200)	800 (400-1325)	0.511
Diuresis (ml/kg/h)	1.13 (0.76-1.85)	0.896 (0.56-1.43)	0.068
Norepinephrine	3 (5.88%)	11 (20.37%)	0.058
Dobutamine	2 (3.92%)	0 (0%)	0.451
Vasodilatation therapy	5 (9.8%)	3 (5.56%)	0.652
			
After eight hours on ICU			
Crystalloids (ml)	1587 ± 371	1528 ± 475	0.485
Colloids (ml)	0 (0-500)	0 (0-250)	0.887
Blood (ml)	0 (0-0)	0 (0-0)	0.746
Fresh frozen plasma (ml)	0 (0-0)	0 (0-0)	0.744
Diuresis (ml/kg/h)	1.18 (0.79-1.89)	1.08 (0.83-1.89)	0.886
Norepinephrine	7 (13.72%)	6 (11.11%)	0.913
Dobutamine	1 (1.96%)	0 (0%)	0.977
Vasodilatation therapy	10 (19.61%)	9 (16.67%)	0.891
Diuretic support	13 (25.49%)	17 (31.48%)	0.643
SOFA (8 hours)	3 (2-5.25)	3 (1-4)	0.474
SOFA (24 hours)	2 (2-4)	3 (1.5-4)	0.541

Perioperative period analyzed only for patients whose intraoperative protocol was carried in full extent. Values are presented as absolute (percentage), mean ± standard deviation or median (interquartile range).

CI, cardiac index; CVP, central venous pressure; HR, heart rate; MAP, mean arterial pressure; N/A, not applicable; SOFA, Sequential Organ Failure Assessment; SVV, stroke volume variation.

**Figure 3:**
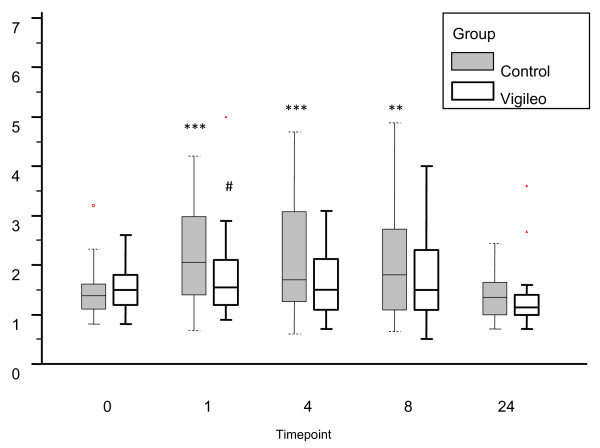
**Serum lactate concentrations (mmol/l)**. ** *P *< 0.01, *** *P *< 0.001 analysis of variance on repeated measurements against baseline; ^# ^*P *< 0.05 Vigileo vs. Control (t-test).

An increase in serum lactate concentration was observed in the Control group compared with baseline at the end of surgery, four and eight hours after operation (*P *< 0.01, ANOVA on repeated measurements with Bonferroni correction). We found no such difference in the Vigileo group. Serum lactate concentration at the end of the operation in the Vigileo group was lower than in the Control group (1.78 mmol/l vs. 2.25 mmol/l; *P *= 0.0252). Arterial pH decreased at the end of operation in both groups compared with baseline and normalized during postoperative period; however, in the Vigileo group the pH at the end of operation was higher (7.37 in the Vigileo vs. 7.35 in the Control groups; *P *= 0.049). A concomitant decrease in base excess and serum bicarbonate from baseline at the end of surgery was observed in both groups but normalized early in the postoperative period, no difference was found between the groups. In comparison with baseline the ScvO _2 _in both groups was higher at the end of surgery and was lower 24 hours after operation (ANOVA on repeated measurement), no difference between the groups was observed. All results are presented in Table 4 and Figure 3.

**Table 4:** Biochemical variables

Variable	Baseline	End of surgery	4 hours postOP	8 hours postOP	24 hours postOP	ANOVA on rpt.m.
**Serum lactate (mmol/l)**						
Vigileo	1.48 ± 0.44	**1.78 ± 0.83**^#^	1.75 ± 0.86	1.85 ± 0.98	1.25 ± 0.52	0.002
Control	1.42 ± 0.43	2.25 ± 1.12***	2.14 ± 1.11***	2.10 ± 1.18**	1.4 ± 0.50	< 0.001
**Arterial pH**						
Vigileo	7.43 ± 0.03	7.37 ± 0.05*** ^#^	7.39 ± 0.04**	7.41 ± 0.05	7.41 ± 0.03*	< 0.001
Control	7.41 ± 0.04	7.35 ± 0.05***	7.38 ± 0.05**	7.40 ± 0.05	7.42 ± 0.04	< 0.001
**Base excess (mmol/l)**						
Vigileo	0.67 ± 1.72	-1.55 ± 1.91***	-0.23 ± 2.19*	0.41 ± 1.8	1.36 ± 2.36	< 0.001
Control	-0.19 ± 2.55	-2.15 ± 2.54***	-0.55 ± 2.44	-0.09 ± 2.64	1.17 ± 2.17	< 0.001
**Serum bicarbonate (mmol/l)**						
Vigileo	24.63 ± 1.81	23.05 ± 1.68***	24.11 ± 2.36	24.65 ± 1.84	25.59 ± 2.59	< 0.001
Control	23.81 ± 2.69	22.67 ± 2.16**	24.04 ± 2.28	24.33 ± 2.57	25.45 ± 2.94	< 0.001
**ScvO2 (%)**						
Vigileo	71.79 ± 6.94	80.18 ± 7.86***	69.43 ± 8.40	68.54 ± 8.23	67.61 ± 6.54*	< 0.001
Control	72.27 ± 6.77	80.04 ± 6.87*	69.00 ± 7.92	69.50 ± 7.84	67.36 ± 7.14**	< 0.001
**Hemoglobin (g/dl)**						
Vigileo	12.3 ± 1.5	10.5 ± 1.1***	11.4 ± 1.5***	11.2 ± 1.5***	10.7 ± 1.3***	< 0.001
Control	12.7 ± 1.6	10.4 ± 1.2***	11.4 ± 1.3***	11.2 ± 1.4***	10.8 ± 1.0***	< 0.001

Perioperative period analyzed only for patients whose intraoperative protocol was carried in full extent; *P < 0.05 **P < 0.01 ***P < 0.001 analysis of variance (ANOVA) on repeated measurements with Bonferroni correction against baseline; ^#^*P*< 0.05 Vigileo vs. Control (t-test); Values are presented as mean ± standard deviation.

PostOP, postoperatively; ScvO2, central venous oxygen saturation.

Results of postoperative outcome are presented in Table 5 on an intention-to-treat basis and also as per protocol analysis. The incidence of organ and infectious complications in the 30-day postoperative period was lower in the Vigileo group (18 patients (30%) vs. 35 (58.3%); *P *= 0.0033; relative risk = 0.514; 95% confidence interval = 0.331 to 0.8) and also the number of complications was significantly diminished (34 vs. 77; *P *= 0.0066). The incidence of severe complications (7 patients (11.7%) vs. 22 (36.7%); *P *= 0.0028; relative risk = 0.318; 95% confidence interval = 0.147 to 0.688) and their number (13 complications vs. 41; *P *= 0.0132) was also lower in the Vigileo group. There was no difference in mortality, hospital and ICU length of stay between groups. Similar results were obtained when analyzing only patients whose optimization was carried out: complication rate was lower in Vigileo (16 patients (31.37%) vs. 32 (59.26%); *P *= 0.0076; relative risk = 0.5294; 95% confidence interval = 0.3335 to 0.8405) as well as their number (32 vs. 73; *P *= 0.0141). Severe complications developed six patients (11.76%) in Vigileo vs. 19 (35.19%) in the Control group (*P *= 0.0097; relative risk = 0.3344; 95% confidence interval = 0.1452 to 0.7701) and their number (12 complications vs. 38; *P *= 0.0274) was also lower in the Vigileo group. In this per protocol group a lower rate of complications was even associated with shorter hospital length of stay in the Vigileo group (9 (8 to 12) vs. 10 (8 to 19); *P *= 0.0421). Again there was no difference in mortality and ICU length of stay.

**Table 5:** Summary of outcomes and complications

Parameters	Vigileo group	Control group	*P *value
Number of patients			
ITT analysis	60	60	
Per protocol analysis	51	54	

Mortality (%)			
ITT	1 (1.67%)	2 (3.33%)	1.0
Per protocol	1 (1.96%)	1 (1.85%)	0.501
Hospital length of stay (days)			
ITT	**9 (8-11.5)**	**10 (8-16)**	**0.0937**
Per protocol	**9 (8-12)**	**10(8-19)**	**0.0421**
ICU length of stay (days)			
ITT	3 (2-5)	3 (0.5-5)	0.789
Per protocol	3 (2-6)	3 (2-5)	0.368
Rehospitalization (ITT only)	2 (3.33%)	6 (10%)	0.272
Morbidity (day 30)			
Patients with complications			
ITT	**18 (30%)**	**35 (58.3%)**	**0.0033**
Per protocol	**16 (31.37%)**	**32 (59.26%)**	**0.0076**
Patient with severe complication(*)			
ITT	**7 (11.7%)**	**22 (36.6%)**	**0.0028**
Per protocol	**6 (11.76%)**	**19 (35.19%)**	**0.0097**
Complications (day 30)			
ITT	**34**	**77**	**0.0066**
Per protocol	**32**	**73**	**0.0141**
Severe complications (day 30) (*)			
ITT	**13**	**41**	**0.0132**
Per protocol	**12**	**38**	**0.0274**
List of complications (ITT only)			
Infectious			
Pneumonia *	4	8	
Sepsis *	1	8	
Intraabdominal infection *	1	4	
Catheter-related bloodstream inf. *	1	8	
Urinary tract infection	3	13	
Wound infection/dehiscence	2	5	
Decubital inf.	1	3	
Cardiovascular			
Arrhythmias (non-life threatening)	3	5	
Arrhythmias (life threatening) *	0	0	
Heart failure/Pulmonary edema *	3	6	
Acute myocardial infarction *	0	1	
Respiratory			
Pulmonary embolism *	0	0	
ALI/ARDS *	0	0	
Ventilator support (incl.postoperative)	3	6	
New onset of ventilator support *	2	4	
Renal			
AKI (without dialysis)	2	4	
Renal failure with dialysis *	1	1	
Thrombotic			
Stroke (including TIA) *	0	1	
Deep vein thrombosis	0	1	
Graft thrombosis	1	3	
Gastro-intestinal			
GIT bleeding	0	0	
GIT obstruction	0	0	
Pancreatitis (edematous/necrotizing *)	2/0	0/0	
Hepatic dysfunction (mild/severe *)	0/0	1/0	

Values are presented as absolute (percentage) or median (interquartile range).

* Complication deemed as severe (life disabling or threatening).

AKI, acute kidney injury; ALI/ARDS, acute lung injury/acute respiratory distress syndrome; GIT, gastro-intestinal tract; ITT, intention to treat analysis; Per protocol, only patients whose intraoperative protocol was carried in full extent; TIA, transient ischemic attack.

When analyzing patients according to developed complications, a higher serum lactate level at the end of surgery (1.5 (1.2 to 2.2) vs. 2 mmol/l (1.4 to 2.8); *P *= 0.0084) and four hours after (1.4 (1 to 2) vs. 2 mmol/l (1.4 to 3.1); *P *= 0.003) was observed in the group with complications. Severe complications were associated with lower CI at the end of surgery (3.7 ± 0.7 vs. 2.95 ± 0.3; *P *= 0.0108). No difference in postoperative ScvO2 or other laboratory and hemodynamic parameters was found. Length of stay in ICU (3 (0 to 4) vs. four days (2 to 6); *P *= 0.0054) and in hospital (8 (7 to 10) vs. 11 days (8 to 16.8); *P *< 0.0001) was shorter in the group without complications.

## Discussion

Intraoperative fluid optimization in high-risk surgical patients undergoing major abdominal surgery using SVV and Vigileo/FloTrac monitor increased hemodynamic stability during operation, decreased lactate concentration at the end of operation and was associated with a lower rate of postoperative complications with a tendency to decrease hospital length of stay.

To our knowledge, this is the first study using a Vigileo/FloTrac monitor in the perioperative setting for guiding fluid management mainly by SVV. Recently a study by Mayer and colleagues [42], looking at a group of surgical patients, was optimized using Vigileo/FloTrac was published with very similar results regarding postoperative complication rates and counts. Also, the same software version (second generation - 1.10) was used. Including Vigileo software version information is of critical importance, as second-generation devices seem to be more accurate according to some studies [31-35] although many controversial results have been presented recently. Reliability of the device was questioned in hemodynamically unstable patients [43], in those with high heart rate variability or when sudden changes in vascular tone occur as in cases of vasoactive drugs bolus administration etc [36,37], or during hepatic surgery [44,45]. Specifically, the influence of systemic vascular resistance alteration on accuracy of the Vigileo monitor is of note and might be a source of possible bias, particularly in patients under general anesthesia. Systemic vascular resistance was measured in the Vigileo group and no significant divergences from normal ranges were observed. Furthermore, the use and dosage of vasopressors was relatively low in our patients. Moreover, a sustained change of hemodynamic parameters was one of the conditions in the decision protocol to minimize these flaws. The aim of our study was to evaluate the clinical utility of this safe and easy-to-use device. Using a new software generation (version 3.0 or higher) would probably enhance the monitor performance, but it was not available at the beginning of our study.

Using dynamic variables including SVV, some possible confounders should be considered. We already mentioned the influence of tidal volume [24,25], heart rhythm [38] and use of vasopressors [26]. We tried to minimize all of these as described by using a fixed tidal volume of 8 ml/kg with PEEP 0.6 kPa and excluding patients with irregular heart rhythm. As mentioned,a sustained rise of SVV above 10% in a period of five minutes was needed to start an intervention in order to exclude a possible bias due to surgical manipulations or other influences. We used the 10% threshold proposed by Manecke [27], which was the best available recommended value for Vigileo/FloTrac at the time of preparing our protocol, although the optimal cut-off value for SVV is still uncertain. A study in cardiosurgical patients [21] proposed a lower target of 9.6%, although other trials in patients undergoing major abdominal surgery [22] offered a more liberal value of 12%. Another study [36] was unable to find a good predictive cut-off value under open abdomen conditions, probably showing that some hidden confounder still exists. These inconclusive results show that a further evaluation of dynamic variables is needed and results of protocols based only on variations itself should be assessed with caution. We used a dynamic change of CI and CVP for decision-making as safety measures to forestall these potential flaws.

Two studies were published using dynamic variables for intraoperative fluid management. Lopes and colleagues [11] demonstrated a significant morbidity reduction using solely pulse pressure variation in the optimization of high-risk surgical patients with results similar to our study and other literature concerning GDT [18,27]. A major limitation of that study was the small number of patients included (17 patients optimized and 16 in the control group). The complications rate was high (75% vs. 41%) compared with our results but the proportional reduction was similar. One possible explanation for this disproportion is a higher number of ASA 4 patients. A second study [46] found no treatment benefit using systolic pressure variation (SPV) in guiding fluid management. The reason for different results could lie in the population studied being much healthier and a relatively liberal use of norepinephrine. As SPV is more influenced by afterload modification than SVV [47], patients in this study could have been, despite vigorous fluid resuscitation, still not optimized. This opinion is supported by a high SVV in the study group (12% in 3rdhour and 11% in 6thhour of protocol) compared with low SPV (7% and 6% in the same time points).

The rate and number of postoperative complications in our study were significantly lower in the interventional group. This reduction corresponds with many GDT trials including the recently published study by Mayer and colleagues [42], where only 20% of GDT patients developed complications compared with 50% in the control group. GDT is generally associated with infusion of larger amounts of colloids and improvement in hemodynamic parameters at the end of surgery. A difference between GDT and control groups are described in some studies, but neither the current trial or the one by Mayer and colleagues brings information about more detailed hemodynamic parameters in control groups whose management was guided with standard care (CVP, MAP etc). This naturally limits interpretation about whether the SVV-guided fluid loading is really the cause of morbidity reduction. Nevertheless, the one of the most probable causes of GDT success is a timely recognition of hemodynamic derangements and prompt intervention for their solution. Such an effect may not lead to a significant difference of hemodynamic variables at the end of surgery, but more probably would manifest in biochemical markers resulting from these derangements.

Biochemical parameters of oxygen debt (serum lactate level, its normalization and low ScvO2 or low mixed venous oxygen saturation (SvO2)) could serve as these markers and are early indicators of unfavorable outcome in critically ill patients [4,48,49]. In our study we observed an increase in serum lactate concentration in the Control group, which was associated with arterial pH decrease and persisted for eight hours postoperatively. The course of ScvO2 values at different time points was similar in both groups with a slight elevation during anesthesia and decrease 24 hours after operation. A difference in arterial serum lactate level between patients with complications and those without was detected. A higher mean lactate 24 hours postoperatively in the study by Lopes and colleagues [11] and Chytra and colleagues [9] showed that GDT decreased lactate level with a possible association to a reduction of infectious complications. A good predictive value of postoperative ScvO2 was found by Pearse and colleagues [48] in the analysis of their GDT study with a proposed cut-off value of 64.4%, and another study [49] found even higher predictive ScvO2 levels of 73%. We were unable to prove any correlation between ScvO2 and postoperative morbidity. A large portion of vascular surgery procedures (above 60% in both groups) probably contributed to this phenomenon. Lactate generated during aortic cross clamping in lower body parts spread after clamp release into systemic circulation. Hemodynamic instability and low intravascular volume during the reperfusion period could delay restitution of normal flow promoting ischemia-reperfusion injury with consequent complications. This may impact on the fidelity of ScvO _2 _and arterial serum lactate levels might be a better predictor of outcome under these circumstances. Lactate-free fluids were used for volume substitution to exclude potential bias and the time of ischemia producing aortic cross-clamping was measured and was comparable between groups.

In contrast to the lower incidence and number of complications a limited impact on the length of stay in the ICU or hospital was found in our study. Only hospital length of stay in those patients whose optimization protocol was carried out was reduced. However, some factors can limit the generalization of these findings. Each institution usually has its own regimens and protocols of ICU and ward care, which can significantly change the length of stay. A 'medical fitness for discharge' was used by some authors [8,13,16] to overcome the problem of social hospitalization and other biases. Discharge criteria were not predefined in our study, which can limit the interpretation of both (hospital and ICU) length of stay parameters. Other possible reasons why the ICU length of stay was similar in both groups, although rate of severe complications was higher in the controls, is a suspected 'overtreatment' of patients without complications (a median of three days on ICU was observed in uncomplicated patients). When comparing other GDT studies the hospital length of stay is similar to our results in both groups [7,8,11,14]. Some other factors such as mobilization of patients or suture removal after laparotomy can limit and influence length of stay on the surgical ward more than medical fitness to discharge. A higher proportion of rehospitalized patients in the control group (3.3% vs. 10%; although not statistically significant) could support this notion. Low mortality counts were observed in both groups in comparison with other authors [11,12,42], but similar to those proposed by POSSUM (Portsmouth modification) and APACHE II scoring systems (both 2.82%). Inclusion of emergency patients [12] and a higher proportion of ASA grade 4 patients [11] or possibly an older population and more complex surgical procedures [42] in other studies can also explain this difference.

The single-center design belongs to major limitations of the trial. The potential bias resulting from institutional standards of care has already been discussed. Also the inclusion of a mixture of surgical procedures could influence our results, because the pathophysiology and causes of complications vary between vascular and non-vascular major abdominal surgery. Our goal was to evaluate the optimization protocol on a nonspecific surgical population usually treated in our institution. We conducted a retrospective analysis of patients undergoing similar surgical procedures and proposed a suitable group size in order to reach the predefined goal of morbidity reduction. A better understanding of observed relations would be possible with a more homogenous population or by a subgroup analysis of a larger sample. Also our study lacks power to show a significant reduction in mortality. On the contrary the extensive reduction in morbidity in such a small population advocates the value of this relatively simple intervention; however, it will have to be proven in a larger multi-center study.

## Conclusions

Optimization of intravascular volume during major abdominal surgery using SVV and Vigileo cardiac output monitor is associated with better intraoperative hemodynamic stability and decrease in serum lactate concentration at the end of surgery. In the postoperative period a significantly lower incidence of complications were found. A larger and multicenter study, optimally using the novel software generation should be performed to confirm results of our study.

## Key messages

• In this study, intraoperative hemodynamic optimization using SVV in high-risk patients undergoing major abdominal surgery was associated with improved hemodynamic stability and reduced serum lactate concentration at the end of surgery.

• In this study, GDT using SVV as an end-point was associated with reduced postoperative complication rates.

## Abbreviations

APACHE: Acute Physiologic and Chronic Health Evaluation; ANOVA: analysis of variance; ASA: American Society of Anesthesiologists'physical status classification; CI: cardiac index; CVP: central venous pressure; FFP: fresh frozen plasma; GDT: goal-directed therapy; MAP: mean arterial pressure; PEEP: positive end-expiratory pressure; POSSUM: Physiological and Operative Severity Score for the Enumeration of Mortality and Morbidity; RBC: red blood cells; ScvO2: central venous oxygen saturation; SOFA: Sequential Organ Failure Assessment; SPV: systolic pressure variation; SVV: stroke volume variation.

## Competing interests

The authors declare that they have no competing interests.

## Authors' contributions

JB, ICH, EK and RP were responsible for the study design. JB, PA, MH, RS and MS were responsible for administering the protocol. JB and ICH provided the data analysis and drafting the manuscript. All authors have given final approval of this version of the manuscript.
